# Cardiovascular sequelae of trastuzumab and anthracycline in long-term survivors of breast cancer

**DOI:** 10.1136/heartjnl-2023-323437

**Published:** 2023-12-16

**Authors:** Claire Glen, Andrew Morrow, Giles Roditi, Tracey Hopkins, Iain Macpherson, Philip Stewart, Mark C Petrie, Colin Berry, Fred Epstein, Ninian N Lang, Kenneth Mangion

**Affiliations:** 1 School of Cardiovascular and Metabolic Health, University of Glasgow, Glasgow, UK; 2 Clinical Research Imaging Facility, NHS Greater Glasgow and Clyde, Glasgow, UK; 3 School of Cancer Sciences, University of Glasgow, Glasgow, UK; 4 Regional Heart and Lung Centre, Golden Jubilee National Hospital, Clydebank, UK; 5 Cardiology, Golden Jubilee National Hospital, Clydebank, UK; 6 School of Engineering and Applied Science, University of Virginia, Charlottesville, Virginia, USA

**Keywords:** Magnetic Resonance Imaging, Drug Monitoring

## Abstract

**Objectives:**

Long-term follow-up of patients treated with trastuzumab largely focuses on those with reduced left ventricular ejection fraction (LVEF) on treatment completion. This study sought to evaluate the prevalence of cardiovascular risk factors, overt cardiovascular disease and cardiac imaging abnormalities using cardiac magnetic resonance (CMR), in participants with normal LVEF on completion of trastuzumab±anthracycline therapy at least 5 years previously.

**Methods:**

Participants with human epidermal growth factor receptor 2-positive breast cancer treated with trastuzumab±anthracycline ≥5 years previously were identified from a clinical database. All participants had normal LVEF prior to, and on completion of, treatment. Participants underwent clinical cardiovascular evaluation, ECG, cardiac biomarker evaluation and CMR. Left ventricular systolic dysfunction (LVSD) was defined as LVEF <50%.

**Results:**

Forty participants were recruited between 15 March 2021 and 19 July 2022. Median time since completion of trastuzumab was 7.8 years (range 5.9–10.8 years) and 90% received prior anthracycline. 25% of participants had LVSD; median LVEF was 55.2% (Q1–Q3, 51.3–61.2). 30% of participants had N-terminal pro-B-type natriuretic peptide >125 pg/mL and 8% had high-sensitivity cardiac troponin T >14 ng/L. 33% of participants had a new finding of hypertension. 58% had total cholesterol >5.0 mmol/L, 43% had triglycerides >1.7 mmol/L and 5% had a new diagnosis of diabetes.

**Conclusions:**

The presence of asymptomatic LVSD, abnormal cardiac biomarkers and cardiac risk factors in participants treated with trastuzumab and anthracycline at least 5 years previously is common, even in those with normal LVEF on completion of treatment. Our findings reinforce the relevance of comprehensive evaluation of cardiovascular risk factors following completion of cancer therapy, in addition to LVEF assessment.

**WHAT IS ALREADY KNOWN ON THIS TOPIC**

- Trastuzumab therapy is associated with a risk of left ventricular systolic dysfunction, with surveillance largely focused on the period during treatment.
- The incidence of cardiac dysfunction in the longer term post-trastuzumab plus anthracycline therapy is not well defined.

**WHAT THIS STUDY ADDS**

- Twenty-five per cent of participants treated with trastuzumab and anthracycline at least 5 years previously had left ventricular systolic dysfunction, measured by cardiac magnetic resonance. Abnormal cardiac biomarkers and hypertension were also common.

**HOW THIS STUDY MIGHT AFFECT RESEARCH, PRACTICE OR POLICY**

- Comprehensive evaluation of cardiovascular risk factors following completion of trastuzumab therapy should be carried out in all patients.
- Our results support risk-stratified guidance of serial cardiac function monitoring.

## Introduction

In 2020, 7.8 million women were alive with a diagnosis of breast cancer in the previous 5 years, making breast cancer the most prevalent cancer globally.^1^ The gene encoding human epidermal growth factor receptor 2 (HER2) is overexpressed in 15–20% of breast cancers and was originally associated with a poor prognosis due to accelerated tumour growth and early metastasis.^2 3^ Development of therapies to inhibit the HER2 signalling pathway, including the monoclonal antibody trastuzumab, has transformed the outlook of HER2-positive breast cancer.^4^

Unfortunately, trastuzumab is associated with a significant risk of cardiac dysfunction. Up to a quarter of patients treated with trastuzumab will develop left ventricular systolic dysfunction (LVSD),^5–7^ defined as an asymptomatic decline ≥10 percentage points in left ventricular ejection fraction (LVEF) to an absolute value <50%, or symptomatic heart failure.^8^ The risk of LVSD is greater in patients who have received an anthracycline.^9^ Trastuzumab-related cardiotoxicity has typically been considered to be a reversible phenomenon which recovers with interruption or cessation of treatment.^10–13^ Therefore, surveillance for LVSD has mainly focused on the period during treatment and there is considerable variation in longer-term follow-up around the world.

The incidence of cardiac dysfunction or heart failure in the longer term post-trastuzumab plus anthracycline therapy is not well defined,^13 14^ with evidence from clinical trials reporting no excess risk of heart failure after the first 2–3 years^15–17^ but observational studies suggesting that the incidence may be up to 20% with a longer risk period.^9 18^ The 2022 European Society of Cardiology (ESC) Cardio-Oncology guidelines^19^ recommend that patients undergo risk assessment to identify those who require cardiovascular surveillance following completion of cancer therapy. Patients deemed to be moderate, high or very high risk should be considered for periodical echocardiographic assessment and all patients should undergo an annual cardiovascular risk assessment.

We aimed to evaluate the prevalence of cardiovascular risk factors, overt cardiovascular disease and cardiac imaging abnormalities in patients with normal LVEF on completion of anti-HER2 therapy±anthracycline, at least 5 years previously using cardiac biomarkers, multiparametric cardiovascular magnetic resonance (CMR) and cardiovascular risk factor assessment.

## Methods

### Study population

We conducted a prospective, observational, cohort study of participants treated at a regional cancer hospital network (West of Scotland Cancer Network, National Health Service, UK). Participants aged >18 years, with HER2-positive breast cancer treated with trastuzumab±anthracycline at least 5 years prior to enrolment were invited to participate. Participants with a standard contraindication to CMR, suspected pregnancy or estimated glomerular filtration rate <30 mL/min/1.73 m^2^ were excluded. A previous diagnosis of heart failure or LVSD on completion of trastuzumab was the exclusion criterion. A total of 40 women were enrolled.

### Data collection

All participants attended for comprehensive history and cardiovascular examination. Baseline data collected included age, body mass index, smoking status, history of cardiovascular disease (prior myocardial infarction, coronary artery bypass grafting or stable angina) or cardiovascular risk factors (hypertension, diabetes mellitus or chronic kidney disease), history of heart failure, atrial fibrillation, valvular heart disease or chronic obstructive pulmonary disease, anthracycline and trastuzumab treatment regimens and cardiovascular medications. ECG and clinic blood pressure measurement were carried out. Blood pressure measurements were taken with the participant seated for at least 5 min prior using an appropriately sized cuff and a clinically validated automatic oscillometric device. If elevated, a second reading was taken 2–3 min later and an average taken. An elevated office systolic blood pressure threshold of >140 mm Hg was made in line with ESC guidelines for the management of arterial hypertension.^20^ Blood samples collected for full blood count, blood chemistry, lipid profile and glycosylated haemoglobin (HbA1c) were processed real time in an accredited National Health Service laboratory. High-sensitivity troponin T (hsTnT) and N-terminal pro-B-type natriuretic peptide (NT-proBNP) samples were centrifuged and frozen at −80°C and subsequently analysed using the Elecsys Troponin T hs (Roche, Basel, Switzerland) and Elecsys proBNP II (Roche, Basel, Switzerland) assays respectively. Thresholds of abnormal values for lipid profile (high-density lipoprotein <1.0 mmol/L, low-density lipoprotein >3.0 mmol/L, triglycerides>1.7 mmol/L) and NT-proBNP (>125 pg/mL) were made on the basis of the relevant ESC guidelines.^21 22^ Thresholds for elevated hsTnT (>14 ng/L, sex specific) and HbA1c (>42 mmol/mol) were made from laboratory reference ranges. Participants were categorised as low, moderate, high or very high risk using the ESC cardiovascular toxicity risk stratification tool for asymptomatic adult cancer survivors (table 1).

**Table 1:** ESC risk categories for asymptomatic adult cancer survivors

Risk category*	Patient characteristics
Very high risk	Very high baseline cardiovascular toxicity risk pretreatment
	Doxorubicin† ≥400 mg/m^2^
	RT >25 Gy MHD‡
	RT >15–25 Gy MHD‡+doxorubicin† ≥100 mg/m^2^
Early high risk (<5 years after therapy)	High baseline cardiovascular toxicity risk
	Symptomatic or asymptomatic moderate-to-severe CTRCD during treatment
	Doxorubicin† 250–399 mg/m^2^
	High-risk HSCT§
Late high risk	RT >15–25 Gy MHD‡
	RT 5–15 Gy MHD¶+doxorubicin† ≥100 mg/m^2^
	Poorly controlled cardiovascular risk factors
Moderate risk	Moderate baseline cardiovascular toxicity risk
	Doxorubicin† 100–249 mg/m^2^
	RT 5–15 Gy MHD¶
	RT <5 Gy MHD**+doxorubicin† ≥100 mg/ m^2^
Low risk	Low baseline cardiovascular toxicity risk and normal end-of-therapy cardiac assessment
	Mild CTRCD during therapy but recovered by the end of cancer
	RT <5 Gy MHD**
	Doxorubicin†, 100 mg/m^2^

*RT risk categorisation based on MHD is recommended over categorisation based on prescribed dose, which may not accurately reflect cardiac radiation exposure. Depending on dose distribution and exposure of specific cardiac substructures (as well as clinical risk factors), the treatment team may judge the patient to belong to a higher risk category. In addition, a patient may be judged to belong to a lower risk category in case only a small part of the heart is exposed to a relatively high prescribed dose.

†Or equivalent.

‡Or prescribed RT ≥35 Gy to a volume exposing the heart if MHD is not available. Note that in this case, the limited information about cardiac exposure does not allow one to distinguish between high-risk and very high-risk categories.

§High-risk HSCT patients: allogenic HSCT; pre-existing CVD or multiple uncontrolled cardiovascular risk factors; cancer treatment history (mediastinal or mantle field radiation, alkylating agents, >250 mg/m^2^ doxorubicin or equivalent); conditioning schemes (total body irradiation, alkylating agents); development of GVHD.

¶Or prescribed RT 15–34 Gy to a volume exposing the heart if MHD is not available.

**Or prescribed RT, 15 Gy to a volume exposing the heart if MHD is not available.

CTRCD, cancer therapy-related cardiac dysfunction; CVD, cardiovascular disease; ESC, European Society of Cardiology; GVHD, graft-versus-host disease; Gy, Gray; HSCT, haematopoietic stem cell transplant; MHD, mean heart dose; RT, radiotherapy.

### CMR imaging and analyses

Participants were scanned using a clinical research-dedicated 3.0 Tesla MRI scanner (MAGNETOM Prisma, Siemens Healthineers) with a standard phased array cardiac surface coil. The protocol included cine imaging, T1 and T2 parametric mapping, DENSE (displacement encoding with stimulated echoes) and late gadolinium enhancement (LGE) sequences. After deidentification, MRI scans were reviewed and reported by an accredited radiologist with >15 years’ experience. Further details are available in the online supplemental appendix 1.

10.1136/heartjnl-2023-323437.supp1 Supplementary data

### Statistical analysis

Baseline characteristics were summarised according to evidence of cardiotoxicity, defined as LVEF <50%. All data are presented as mean±SD or median (25th–75th percentiles, Q1–Q3) according to distribution. Categorical variables are presented as numbers and percentages. Student’s t-test or Χ^2^ tests were used to determine the association of baseline characteristics with the development of cardiotoxicity. Χ^2^ test was used to assess the association between ESC cardiovascular toxicity risk stratification tool and the development of cardiotoxicity.

All analyses were performed using STATA V.17 software (StataCorp 2021. Stata Statistical Software; College Station, Texas, USA). Statistical significance was defined as two-tailed p<0.05 for all tests. Please see online supplemental file 1 for sample size calculation.

## Results

A total of 43 women were enrolled. Three women were unable to complete the CMR protocol due to claustrophobia and were excluded from analysis. Mean age was 62 years±10 years. Median time since completion of trastuzumab was 7.8 years (range 5.9–10.8 years). Thirty-six participants (90%) received prior anthracycline therapy; 35 participants received three to six cycles of epirubicin and the remaining patient received four cycles of doxorubicin. In all cases, trastuzumab was initiated following completion of adjuvant anthracycline. Twenty-three participants (58%) also received radiotherapy (43% left sided). Clinical details are summarised in table 2.

**Table 2:** Baseline clinical demographics

	N=40
Age (years)	62 (10)
BMI	26.8 (23.6–31.0)
History	
MI	0 (0%)
HF	0 (0%)
PVD	0 (0%)
CVD	0 (0%)
COPD	1 (3%)
Diabetes	0 (0%)
Valvular heart disease (non-severe)	3 (8%)
AF	1 (3%)
HTN	10 (25%)
Smoking	8 (20%)
Current	1 (3%)
Previous	7 (17%)
Baseline ICOS risk score	
Low	4 (10.0%)
Medium	23 (57.5%)
High	13 (32.5%)
Adult cancer survivor risk category	
Low	2 (5%)
Moderate	18 (45%)
Early high	19 (48%)
Very high	1 (2%)
Trastuzumab cumulative dose (mg)	7318.5 (6184.5–8494.0)
Number of trastuzumab cycles	16±3
Number of patients treated with anthracyclines prior to trastuzumab	36 (90%)
Cumulative anthracycline dose in doxorubicin equivalent (mg/m^2^)	175 (161–195)
Number of anthracycline cycles	3±1
Radiotherapy	23 (58%)
Left sided	10 (43%)
Right sided	13 (57%)
Prescribed medication	
Aspirin	0 (0%)
Beta-blocker	4 (7%)
ACEi/ARB	10 (17%)
Statin	9 (16%)
Calcium channel blocker	5 (9%)
Thiazide diuretic	3 (5%)
Anticoagulant	1 (2%)
End-of-treatment LVEF measured by echo (%)	64 (7.8)

Data are presented as mean±SD or median (IQR) for continuous measures (dependent on distribution) and n (%) for categorical measures.

ACEi, ACE inhibitor; AF, atrial fibrillation; ARB, angiotensin receptor blocker; BMI, body mass index; COPD, chronic obstructive pulmonary disease; CVD, cardiovascular disease; HF, heart failure; HTN, hypertension; ICOS, International Cardio-Oncology Society; LVEF, left ventricular ejection fraction; MI, myocardial infarction; PVD, peripheral vascular disease.

### Cardiovascular risk assessment

Ten participants (25%) had a history of hypertension, seven participants (17%) were previous smokers and one participant (3%) was a current smoker. At baseline, 17% of participants were prescribed an ACE inhibitor or angiotensin receptor blocker (ARB), 16% a statin, 9% a calcium channel blocker and 7% beta-blocker therapy. There was no significant difference in prescription of beta-blocker, ACE inhibitor/ARB or statin between those with reduced left ventricular function and those with normal LVEF.

ECG in all participants showed sinus rhythm with normal PR, QRS and QTc intervals with no T wave or ST segment changes. Twenty-one participants were noted to have an office systolic blood pressure >140 mm Hg, of whom 8 had a history of hypertension and the remaining 13 participants were not known to be hypertensive previously. Twenty-three participants (58%) had a total cholesterol >5.0 mmol/L and 17 participants (43%) had triglyceride level >1.7 mmol/L. Two participants (5%) had elevated HbA1c levels consistent with a new diagnosis of diabetes mellitus. There were no significant differences in cardiac risk factors between those with reduced left ventricular systolic function and those with normal LVEF. Results of cardiovascular risk assessment are shown in table 3.

**Table 3:** Cardiovascular risk assessment

	N=40
Systolic BP (mm Hg)	144 (±19)
Diastolic BP (mm Hg)	80 (±11)
ECG parameters	
Heart rate	72 (±12)
Sinus rhythm	40 (100%)
PR interval (ms)	157 (144–167)
QRS duration (ms)	76 (74–84)
QTc interval (ms)	429 (415.5–440.5)
Blood results	
Total cholesterol (mmol/L)	5.9 (±1.3)
HDL (mmol/L)	1.6 (±0.4)
Total cholesterol:HDL ratio	3.4 (2.8–4.3)
Triglycerides (mmol/L)	1.5 (1.0–2.4)
HbA1c (mmol/mol)	37 (36–41)
Total cholesterol >5.0 mmol/L	23 (58%)
Total cholesterol:HDL ratio >4	11 (28%)
HDL <1.0 mmol/L	1 (3%)
LDL >3.0 mmol/L	19 (48%)
Triglycerides >1.7 mmol/L	17 (43%)
HbA1c >42 mmol/mol	2 (5%)

Data are presented as mean±SD or median (IQR) for continuous measures (dependent on distribution) and n (%) for categorical measures.

BP, blood pressure; HbA1c, glycosylated haemoglobin; HDL, high-density lipoprotein; LDL, low-density lipoprotein.

### Cardiovascular magnetic resonance

The median LVEF was 55.2% (Q1–Q3, 51.3–61.2). Ten participants (25%) had an LVEF <50%. Participants with LVEF <50% had significantly larger indexed left ventricular end-diastolic volume (LVEDV) and left ventricular end-systolic volume (LVESV) than those with normal LVEF; LVEDV 80.6 mL/m^2^ (Q1–Q3, 68.6–91.1) vs 66.7 mL/m^2^ (Q1–Q3, 60.5–80.2), p=0.034 and LVESV 40.9 mL/m^2^ (Q1–Q3, 34.8–49.6) vs 28.9 mL/m^2^ (Q1–Q3, 23.3–34.9), p<0.001. Global longitudinal strain (GLS) measured by both feature tracking (FT) and DENSE methods was significantly better in participants with normal LVEF compared with those with LVEF <50%; GLS by FT −16.2% (Q1–Q3, −17.2 to −14.8) vs −13.7% (Q1–Q3, −16.4 to −11.8), p=0.049 and GLS by DENSE −8.3% (Q1–Q3, −9.5 to −5.6) vs −5.3% (Q1–Q3, −7.8 to −3.0), p=0.034. There was no significant difference in T1, T2 or extracellular volume (ECV) values between those with normal LVEF and those with LVEF <50%. No participants had LGE. Summary of CMR findings is shown in table 4.

**Table 4:** Cardiac MRI findings

	All participants	No cardiotoxicity	Cardiotoxicity	P value	Reference ranges^25 27 28^
	N=40	N=30	N=10		
LV ejection fraction (%)	55.2 (51.3–61.2)	58.5 (54.5–61.6)	49.2 (45.5–49.9)	**<0.001**	51–70
LV end-diastolic volume, indexed to BSA (mL/m^2^)	69.8 (62.9–82.1)	66.7 (60.5–80.2)	80.6 (68.6–91.1)	**0.034**	54–94
LV end-systolic volume, indexed to BSA (mL/m^2^)	30.3 (25.4–37.4)	28.9 (23.3–34.9)	40.9 (34.8–49.6)	**<0.001**	19–40
LV myocardial mass, indexed to BSA (g/m^2^)	38.6 (35.9–44.2)	38.5 (36.0–41.6)	44.6 (35.8–50.3)	0.22	29–55
RV ejection fraction (%)	57.7 (53.2–62.9)	59.6 (54.7–63.7)	54.9 (51.1–57.6)	0.061	47–68
RV end-diastolic volume, indexed to BSA (mL/m^2^)	65.0 (56.8–72.2)	64.3 (56.4–72.0)	66.3 (59.7–77.9)	0.33	53–99
RV end-systolic volume, indexed to BSA	26.7 (22.8–32.0)	25.5 (21.3–31.6)	30.4 (28.1–33.0)	0.053	17–46
LV global circumferential strain by FT (%)	−18.8 (−20.7 to −17.6)	−19.3 (−21.1 to −18.4)	−17.2 (−17.9 to −15.3)	**0.002**	−24 to −13
LV global longitudinal strain by FT (%)	−16.0 (−17.1 to −13.7)	−16.2 (−17.2 to −14.8)	−13.7 (−16.4 to −11.8)	**0.049**	−27 to −11
LV global circumferential strain by DENSE (%)	−18.1 (−20.0 to −17.1)	−18.2 (−20.4 to −17.2)	−17.5 (−18.1 to −15.3)	0.065	−24 to −12
LV global longitudinal strain by DENSE (%)	−7.3 (−9.5 to −5.0)	−8.3 (−9.5 to −5.6)	−5.3 (−7.8 to −3.0)	**0.034**	−19 to −11
LV global T1 (ms)	1205.1 (1174.7–1222.5)	1209.2 (1180.1–1223.6)	1200.4 (1167.3–1208.9)	0.18	1107–1234
Global ECV (%)	27.1 (25.8–28.3)	27.6 (26.5–28.6)	26.9 (18.7–27.5)	0.17	>27.4
LV global T2 (ms)	40.7 (39.3–42.5)	41.1 (39.3–42.5)	39.5 (38.9–42.0)	0.42	35–44

Data are presented as median+IQR.

Bold values are statistically signficant.

BSA, body surface area; DENSE, displacement encoding with stimulated echoes; ECV, extracellular volume; FT, feature tracking; LV, left ventricular; RV, right ventricular.

The majority of participants (90%) received both trastuzumab and anthracycline therapies. Of the four participants who received trastuzumab only, none had an LVEF <50% by CMR. Ten participants (25%) had left-sided radiotherapy. Of these, two participants (20%) had LVEF <50% (p=0.67).

### Biomarkers

Thirty per cent of participants had NT-proBNP concentration >125 pg/mL. Median NT-proBNP was 76.8 pg/mL and there was no significant difference between those with normal LVEF and those with LVEF <50% (75.8 pg/mL (Q1–Q3, 49.4–126.9) vs 89.0 pg/mL (Q1–Q3, 56.7–299.0) respectively, p=0.39). Eight per cent of participants had high-sensitivity cardiac troponin T (hs-cTNT) level >14 ng/L. Median hs-cTNT was 3 ng/L with no difference between normal LVEF and those with LVEF <50% (3.0 ng/L (Q1–Q3, 3.0–5.4) vs 3.0 ng/L (Q1–Q3, 3.0–6.3), p=1.00).

### Adult cancer survivor risk score

The majority of participants in this cohort fell into the ‘moderate’ and ‘early high’ risk categories using the ESC cardiovascular toxicity risk stratification tool for asymptomatic adult cancer survivors who, in line with ESC guidance, would be considered for periodical surveillance with echocardiography. Five per cent were categorised as ‘low’ risk and would not have been recommended to have echocardiography follow-up. One participant was ‘very high’ risk. In participants with an LVEF <50%, 20% were ‘moderate’ risk and 80% were ‘early high’ risk (table 5). There was no significant relationship between risk score and LVEF <50%, χ^2^ (4.76, N=40) p=0.12.

**Table 5:** Adult cancer survivor risk category

	All participants	No cardiotoxicity	Cardiotoxicity
Low	2 (5%)	2 (6.7%)	0
Moderate	18 (45%)	16 (53.3%)	2 (20%)
Early high	19 (48%)	11 (36.7%)	8 (80%)
Very high	1 (2%)	1 (3.3%)	0

## Discussion

In this cohort of women with HER2-positive breast cancer treated with trastuzumab±anthracycline at least 5 years prior, and with normal left ventricular systolic function on completion of therapy, 25% were found to have LVSD by CMR imaging. Participants with reduced LVEF also had significantly larger indexed left ventricular volumes (systole and diastole) and worse GLS indices. New findings of elevated office blood pressure, elevated lipids and elevated NT-proBNP were also common.

Cardiotoxicity associated with trastuzumab and anthracyclines is well recognised. However, most studies evaluating this have been in the earlier phase post-completion of cancer therapy or have evaluated retrospective registry data. This has the potential to limit understanding of the prevalence of asymptomatic late manifestations of cardiotoxicity. To the best of our knowledge, our study is the first to assess cardiotoxicity greater than 5 years post-trastuzumab and anthracycline therapy using a comprehensive CMR protocol, cardiac biomarkers and cardiovascular risk factor assessment. A previous study prospectively assessed left ventricular and right ventricular systolic function using CMR in patients treated with anthracycline and/or trastuzumab at predefined time points up to 60 months.^13^ That study demonstrated a temporary decline in LVEF at 3 months and right ventricular ejection fraction at 12 months which improved to baseline at 60 months and therefore concluded that echocardiographic or CMR follow-up beyond 12 months post-commencement of anthracycline or trastuzumab may not be necessary. It should be noted that only 26 of their initial cohort of 46 returned for follow-up assessment at 60 months and of these, only 38% had received trastuzumab. In contrast, 25% of participants in our cohort had an LVEF <50% with corresponding larger left ventricular volumes and impairment of GLS assessed by two methods and with CMR carried out greater than 5 years post-treatment completion. The majority of participants in our cohort received both trastuzumab and anthracycline therapy with only 10% receiving trastuzumab alone. Given this small number, we are unable to comment on the effect of trastuzumab alone on LVEF and our results represent the effect of trastuzumab plus anthracycline. Notably, there was no significant difference in T1 and T2 relaxation times between those with normal LVEF and reduced LVEF and no participants had LGE. Therefore, while the absence of abnormalities does not provide specific clues to the aetiology of left ventricular impairment in these participants, it also provides further evidence that CMR features of fibrosis and scar are not typically associated with post-trastuzumab LVSD. In patients who present with LVSD post-trastuzumab and anthracycline therapy and who have CMR features of cardiac fibrosis or scar, alternative or other contributing causes of LVSD should be considered.^23 24^ In our cohort, GLS measured by DENSE was reduced in both those with reduced and normal LVEF when compared with a cohort of healthy individuals,^25^ which may suggest subclinical left ventricular dysfunction in patients with normal LVEF. These results require validation in larger cohort studies.

In our cohort, there was a high prevalence of impaired left ventricular systolic function despite a relatively high use of cardiovascular medications at baseline. These medications were principally used for the treatment of hypertension and not for LVSD as all participants had normal LVEF prior to, and on completion of, trastuzumab therapy. While there was no significant difference in the development of LVSD in those with a history of hypertension in comparison with those without, it remains possible that hypertension may contribute to post-trastuzumab and anthracycline LVSD as a component of a multifactorial pathophysiological process. We carried out a comprehensive cardiovascular risk factor assessment and participants were grouped according to the ESC cardiovascular toxicity risk stratification tool for asymptomatic adult cancer survivors.^19^ This tool is used following the completion of treatment to determine those patients who require ongoing long-term follow-up (beyond the first 12 months) and makes recommendations for periodical echocardiographic assessment based on risk category. No participants in the low risk category developed LVSD, although with very small numbers in that group. Our results therefore support the ESC guideline recommendations^19^ that left ventricular assessment should be considered in patients who are moderate risk or greater when this risk score is used. We hope that our study provides further evidence regarding follow-up of this patient cohort which will hopefully inform future guidelines.

Although the guidelines recommend echocardiographic assessment, CMR with GLS measurement may be superior in detecting subclinical changes with reduced interobserver variability due to better endocardial border definition and no geometric assumptions in calculating left ventricular function. Tissue characterisation with T1 and T2 maps, ECV and LGE assessment available with CMR is also valuable in assessing for alternative causes of LVSD. While echocardiography is likely to remain the primary imaging modality used for the assessment of left ventricular systolic function in patients undergoing or post anti-cancer therapies, CMR is a very valuable tool. This is particularly relevant in patients with borderline function or suboptimal imaging in whom CMR gives a more accurate assessment of cardiac function.^26^

ESC guidelines also recommend annual cardiovascular risk assessment including measurement of NT-proBNP and cardiovascular risk factor management in all patients treated with a potentially cardiotoxic anti-cancer therapy. This is not consistently done in clinical practice and is likely to vary considerably between healthcare systems and internationally. However, our results highlight its importance. To the best of our knowledge, none of the participants in this study were being monitored regularly by their primary care provider specifically for ‘cancer survivorship’ reasons. Fifty-three per cent of participants in our cohort had an office systolic blood pressure measurement greater than 140 mm Hg. Of these, 38% had a pre-existing diagnosis of hypertension which was not adequately controlled. All participants with systolic blood pressure >140 mm Hg were advised to attend their primary care provider for repeated blood pressure measurements. A large proportion also had elevated total cholesterol and triglycerides. Thirty per cent of participants had an elevated NT-proBNP >125 pg/mL which is the threshold used for the diagnosis of heart failure. However, in our cohort, elevated NT-proBNP was not significantly associated with impaired LVEF which casts some doubt over the utility of biomarker-based screening for LVSD in this population. A lower NT-proBNP threshold for screening for LVSD may be more appropriate in this group. In our cohort, ECG was not sensitive for LVSD and therefore cannot be relied upon for screening in isolation but remains a valuable test for overall cardiovascular assessment. The importance of surveillance and cardiovascular risk factor management following the completion of treatment needs to be reinforced and implemented in order to reduce long-term cardiovascular complications of anti-cancer therapies in the rapidly growing population of cancer survivors.

### Study limitations

While our study included a relatively small sample size, which limits certain comparisons, it is, to the best of our knowledge, the largest long-term CMR follow-up study after trastuzumab. Although all participants had normal LVEF on completion of therapy, this was measured by echocardiography and it is recommended that imaging surveillance should generally be performed using the same technique. We elected to use CMR rather than echocardiography in our study due to its improved accuracy and reproducibility. Therefore, our assessments focus on the presence or absence of systolic dysfunction at a single time point rather than making an assessment of the changes in LVEF between starting treatment and long-term follow-up. Given the historical nature of treatment, data on the mean heart dose for participants receiving radiotherapy were not available from patient records and so laterality and prescribed dose were used for risk stratification. Blood pressure measurements were made in clinic only with no home blood pressure measurements available. In the event of elevated clinic blood pressure, then an average of multiple readings was taken but we are unable to rule out white coat hypertension. We did not include a non-cancer treatment-exposed age, sex and disease-matched control group; therefore, there is no true comparator to the treatment group.

## Conclusions

The presence of asymptomatic left ventricular systolic impairment, abnormal cardiac biomarkers and cardiac risk factors in participants treated with trastuzumab and anthracycline at least 5 years previously is common, even in those with normal LVEF on completion of treatment. Our findings reinforce the relevance of comprehensive evaluation of cardiovascular risk factors following completion of cancer therapy, in addition to risk-stratified guidance of serial LVEF monitoring.

## Data Availability

All data relevant to the study are included in the article or uploaded as supplemental information. Data access requests should be initially submitted by email to the Chief Investigator.
