# Supplementary material for: Cardiovascular sequelae of trastuzumab and anthracycline in long-term survivors of breast cancer
Source: Heart. 2023 Dec 16;110(9):650–6. doi: 10.1136/heartjnl-2023-323437 (PMC11041571; doi:10.1136/heartjnl-2023-323437)
Supplement: Supplementary data [file heartjnl-2023-323437supp001.pdf]

Cardiovascular Sequalae of Trastuzumab and Anthracycline in Long-Term Survivors of Breast Cancer

Supplemental Material

Supplemental appendix 1…………………………………………………………………………………………… page 2

References………..page 4

Supplemental Appendix 1

Balanced steady-state free precession (SSFP) sequences were used to acquire ventricular cine imaging in three long axis planes (2, 3 and 4 chamber), followed by a short axis stack from the apex to the atrio-ventricular ring, each with 30 phases, for assessment of cardiac function. Three left ventricular short axis (basal, mid and apical) and one orthogonal long axis longitudinal relaxation time (T1, spin–lattice relaxation time constant in milliseconds) motion-corrected, optimized, modified Look-Locker inversion recovery sequences[1,2] were acquired. A short axis stack of T2 prep SSFP[3] (T2, spin–spin relaxation time constant in milliseconds) maps and orthogonal long axis views were acquired, followed by an automated exponential fit for each pixel after respiratory motion correction. Global myocardial extracellular volume (ECV) fraction was analysed by manually contouring LV endocardial and epicardial myocardium and LV blood pool in a single short axis mid-LV slice in both pre- and post-contrast T1 maps. Global extracellular volume fraction was then calculated from pre- (native) and post-contrast myocardial and blood pool T1 values, together with a hematocrit taken on the same day[4]. Feature-tracking strain analysis[5] was assessed using manually contoured LV endocardial and epicardial borders from the short-axis stack and three long-axis (horizontal long axis, vertical long axis and left ventricular outflow tract) cine images in the LV end-diastolic phase (the reference phase). Displacement encoding with stimulated echoes (DENSE) sequences[6] were acquired in three short-axis (basal, mid-ventricular, apical) and three long-axis (horizontal long axis, vertical long axis and left ventricular outflow tract) to assess longitudinal and circumferential strain. Late gadolinium enhancement images, including three long axis acquisitions and a short axis stack, were acquired 10–15minutes after intravenous injection of 0.15mmol kg^{-1} of gadolinium using segmented phase-sensitive inversion recovery sequences.

Glen C, *et al. Heart* 2024; 110:650–656. doi: 10.1136/heartjnl-2023-323437

Segmentation of the LV myocardium was performed semi-automatically after endo- and epicardial contours were drawn, using the anterior right ventricular insertion point as reference. Spatio-temporal phase unwrapping was then carried out on the LV myocardium pixels, and displacement vectors were calculated[7,8] . Lagrangian strain was computed from these displacements and then projected into radial, circumferential (or longitudinal in long axis acquisitions) directions relative to the left ventricular centre of mass. Data was exported as text files.

After deidentification, MRI scans were reviewed and reported by an accredited radiologist (G.R. with >15 years of image analysis experience) and a single image analyst (K.M. with 10 years of image analysis experience). Ventricular volumes, mass, ejection fraction and motion-corrected T1 and T2 sequences were analysed using dedicated software (cvi42 software (version 5.10, Circle Cardiovascular)) by K.M. DENSE data was analysed off-line using a program written in Matlab (Mathworks, UK)[9] by CG.

The following sample size calculation was used for this study. Normal cardiac MRI-derived left ventricular ejection fraction (LVEF) in women is 61% with a standard deviation of 5%. This is derived from reference ranges provided by a biobank of healthy volunteers [10]. The derived incidence of LV dysfunction (LVEF < 50%) in this healthy cohort is predicted to be 1.4%. Assuming the null hypothesis rate of LV dysfunction to be 1.4%, a sample size of 40 will give 85% power at the 5% significance level to detect a rate of LV dysfunction (defined as LVEF <50%) of 10% in our study cohort.

Glen C, *et al. Heart* 2024; 110:650–656. doi: 10.1136/heartjnl-2023-323437

References

1. Rauhalammi SMO, Mangion K, Barrientos PH, et al. Native myocardial longitudinal (T_1) relaxation time: Regional, age, and sex associations in the healthy adult heart. Journal of Magnetic Resonance Imaging. 2016 Sep;44(3):541–8.

2. Messroghli DR, Radjenovic A, Kozerke S, Higgins DM, Sivananthan MU, Ridgway JP. Modified Look-Locker inversion recovery (MOLLI) for high-resolutionT1 mapping of the heart. Magn Reson Med. 2004 Jul;52(1):141–6.

3. Giri S, Chung YC, Merchant A, et al. T2 quantification for improved detection of myocardial edema. Journal of Cardiovascular Magnetic Resonance. 2009 Dec 30;11(1):56.

4. Haaf P, Garg P, Messroghli DR, Broadbent DA, Greenwood JP, Plein S. Cardiac T1 Mapping and Extracellular Volume (ECV) in clinical practice: a comprehensive review. Journal of Cardiovascular Magnetic Resonance. 2017 Jan 30;18(1):89.

5. Liu H, Wang J, Pan Y, Ge Y, Guo Z, Zhao S. Early and Quantitative Assessment of Myocardial Deformation in Essential Hypertension Patients by Using Cardiovascular Magnetic Resonance Feature Tracking. Sci Rep. 2020 Feb 27;10(1):3582.

6. Mangion K, Clerfond G, McComb C, et al. Myocardial strain in healthy adults across a broad age range as revealed by cardiac magnetic resonance imaging at 1.5 and 3.0T: Associations of myocardial strain with myocardial region, age, and sex. Journal of Magnetic Resonance Imaging. 2016 Nov;44(5):1197–205.

7. Spottiswoode BS, Zhong X, Hess AT, et al. Tracking Myocardial Motion From Cine DENSE Images Using Spatiotemporal Phase Unwrapping and Temporal Fitting. IEEE Trans Med Imaging. 2007 Jan;26(1):15–30.

8. Spottiswoode BS, Zhong X, Lorenz CH, Mayosi BM, Meintjes EM, Epstein FH. Motion-guided segmentation for cine DENSE MRI. Med Image Anal. 2009 Feb;13(1):105–15.

9. Gilliam AD, Epstein FH. Automated Motion Estimation for 2-D Cine DENSE MRI. IEEE Trans Med Imaging. 2012 Sep;31(9):1669–81.

10. Petersen SE, Aung N, Sanghvi MM, et al. Reference ranges for cardiac structure and function using cardiovascular magnetic resonance (CMR) in Caucasians from the UK Biobank population cohort. Journal of Cardiovascular Magnetic Resonance. 2017 Feb 3;19(1).

Glen C, *et al. Heart* 2024; 110:650–656. doi: 10.1136/heartjnl-2023-323437
